# Successful Treatment of Human Plague with Oral Ciprofloxacin

**DOI:** 10.3201/eid2303.161212

**Published:** 2017-03

**Authors:** Titus Apangu, Kevin Griffith, Janet Abaru, Gordian Candini, Harriet Apio, Felix Okoth, Robert Okello, John Kaggwa, Sarah Acayo, Geoffrey Ezama, Brook Yockey, Christopher Sexton, Martin Schriefer, Edward Katongole Mbidde, Paul Mead

**Affiliations:** Uganda Virus Research Institute, Entebbe, Uganda (T. Apangu, J. Abaru, G. Candini, H. Apio, F. Okoth, R. Okello, J. Kaggwa, S. Acayo, G. Ezama, E.K. Mbidde);; Centers for Disease Control and Prevention, Fort Collins, Colorado, USA (K. Griffith, B. Yockey, C. Sexton, M. Schriefer, P. Mead)

**Keywords:** plague, *Yersinia pestis*, human, treatment, ciprofloxacin, vector-borne infections, infectious diseases, bacterial infection, FDA approval, bacteria, zoonoses

## Abstract

The US Food and Drug Administration recently approved ciprofloxacin for treatment of plague (*Yersina pestis* infection) based on animal studies. Published evidence of efficacy in humans is sparse. We report 5 cases of culture-confirmed human plague treated successfully with oral ciprofloxacin, including 1 case of pneumonic plague.

Plague is a life-threatening zoonotic disease caused by *Yersinia pestis*. Zoonotic foci exist on several continents; however, resource-poor areas in sub-Saharan Africa account for most human cases (*1*). The pathogenesis of plague involves facultative intracellular infection of host macrophages, followed by fulminant extracellular growth and bacteremia (*2*). In the absence of effective antimicrobial drug treatment, bubonic plague is fatal in ≈50% of cases and pneumonic plague in >90% (*1*,*3*).

Drugs approved by the US Food and Drug Administration (FDA) for treatment of plague include streptomycin and doxycycline. Streptomycin is bactericidal but rarely used because of limited availability and serious toxicities. Doxycycline is bacteriostatic and lacks concentration-dependent activity or a postantibiotic effect, which might limit its efficacy for serious *Y. pestis* infections (*4*). Nevertheless, low cost and oral dosing have made doxycycline a first-line treatment in several countries (*5*,*6*). Fluoroquinolones, include ciprofloxacin, have recently been approved by the FDA for treatment of plague based on animal and in vitro studies (*4*,*7*,*8*). Clinical experience with these agents, however, is limited (*1*,*5*).

During 2011–2014, patients with suspected plague seen at 6 clinics and 2 hospitals in the West Nile region of Uganda were offered enrollment in an open-label study evaluating the safety and efficacy of ciprofloxacin for treatment of plague. Patients were excluded if they were pregnant, <8 years of age, considered too ill to receive oral treatment, or had received antimicrobial drug treatment in the preceding 7 days. After written consent was obtained, diagnostic samples were collected and oral ciprofloxacin administered for 10 days at a weight-calibrated dosage of ≈15 mg/kg twice daily (range 13–17 mg/kg), with a maximum dose for adults of 750 mg twice daily. Diagnostic samples were cultured on sheep blood agar and suspect isolates confirmed by bacteriophage lysis (*9*). Patients were monitored daily during treatment, and clinical outcome was assessed 14–21 days after initial evaluation. Because of simultaneous prevention efforts and lower than expected enrollment, the study was terminated early. The study was approved by Institutional Review Boards at the Uganda Virus Research Institute, the Uganda National Council for Science and Technology, and the US Centers for Disease Control and Prevention.

Five patients with culture-confirmed plague were enrolled and treated with oral ciprofloxacin (Table). Median patient age was 27 years (range 10–52 years); median time between illness onset and enrollment was 4 days (range 1–7 days). Four patients had bubonic plague, with *Y. pestis* isolated from bubo aspirates or blood cultures. The fifth patient, a 13-year-old boy, had pneumonic plague as indicated by hemoptysis, patchy bilateral infiltrates on chest radiograph, and *Y. pestis* isolated from sputum. The illness had evolved over 6 days, a clinical course suggestive of secondary rather than primary pneumonic plague (*3*); the primary focus of infection was not identified.

**Table:** Demographic and clinical characteristics of 5 patients with culture-confirmed plague (*Yersina pestis* infection) who were treated successfully with oral ciprofloxacin, Uganda, 2011–2014

Patient no.	Age, y/sex	Length of illness, d*	Symptoms	Laboratory evidence	Ciprofloxacin dose, mg†
1	10/F	7	Fever, left axillary bubo	Bubo, blood cultures positive	250
2	52/F	4	Fever, right axillary bubo	Bubo, blood cultures positive	650
3	27/F	1	Fever, left inguinal bubo	Bubo, blood cultures positive	750
4	36/M	1	Fever, left axillary bubo	Blood culture positive	625
5	13/M	6	Fever, chest pain, cough, blood-tinged sputum	Sputum culture positive, blood culture negative	375

*At time treatment was sought. †Orally, twice daily; ≈15 mg/kg bodyweight with a maximum of 750 mg.

Three patients were admitted and 2 treated as outpatients. In addition to ciprofloxacin, all received acetaminophen, and 2 received a bolus of normal saline. All became afebrile within 2 days. At 14 days, all had been discharged and returned to their normal activities. The 13-year-old boy with culture-confirmed pneumonic plague reported mild, nonproductive cough, but no complications were identified.

Fluoroquinolones have pharmacokinetic properties that make them attractive for treatment of plague, including bactericidal activity, good oral bioavailability, excellent tissue penetration, and an established safety record (*8*,*10*). In vitro assays suggest that ciprofloxacin is comparable to streptomycin and superior to doxycycline or gentamicin for killing of intracellular *Y. pestis* (*4*), and efficacy has been demonstrated in rodent and nonhuman primate models (*8*). Along with FDA approval, our results add to growing clinical experience (*5*) and support the broader use of oral ciprofloxacin for treatment of human plague, especially in resource-poor areas where intravenous treatment is limited.
